# Isosorbide-Based Acrylic Pressure-Sensitive Adhesives Through UV-Cured Crosslinking with a Balance Between Adhesion and Cohesion

**DOI:** 10.3390/polym16223178

**Published:** 2024-11-14

**Authors:** Jiajie Lv, Qingjun Wang, Hongfeng Xie

**Affiliations:** MOE Key Laboratory of High Performance Polymer Materials and Technology, School of Chemistry and Chemical Engineering, Nanjing University, Nanjing 210023, China; 522022240017@smail.nju.edu.cn

**Keywords:** isosorbide, bio-based pressure-sensitive adhesives, UV-curing, failure mode, cohesion

## Abstract

The development of sustainable pressure-sensitive adhesives (PSAs) from natural biomass resources has attracted increasing attention owing to their non-toxic, biocompatible, and biodegradable features. In this study, a bio-based acrylic PSA with tunable adhesion and cohesion was synthesized by a selective chemical modification of isosorbide-5-acrylate (IA) and its copolymerization with butyl acrylate and acrylic acid through UV-curing crosslinking. During the UV-curing process, the synthesized isosorbide diacrylate ester (IDAE) served as the crosslinker, effectively improving the crosslinking degree of PSA. The impact of IA and IDAE on the mechanical properties of PSA was studied. Moreover, to achieve a balance between adhesion and cohesion, the optimal composition was identified. The addition of IA significantly enhances the stiffness of PSA. Furthermore, the combined effect of IA and IDAE improves the overall adhesion properties of the PSA. The optimal bio-based PSA demonstrates a peel force of 13.9 N/25 mm and a persistent time of 6820 min, promising to replace traditional petroleum-based PSAs.

## 1. Introduction

Pressure-sensitive adhesives (PSAs) can rapidly adhere to various material surfaces under minimal external pressure during a short period without water, solvents, or heat energy [1,2,3,4,5]. PSAs have found extensive applications in label production, medical care, office automation, electronic device assembly, and automotive and construction industries [6,7,8,9]. PSAs are flexible polymers, such as acrylates, rubbers, silicones, and polyurethane, with permanent tack [10,11,12,13]. Among all these PSAs, petroleum-based acrylate is the most commonly used one.

Due to the continuous demand for sustainability and ever-increasing regulations, developing sustainable PSAs from natural biomass resources has received massive interest in recent years [14,15,16]. For acrylic PSAs, bio-based acrylic monomers are of two types: those from biological sources, such as acrylic acid (AA) from fermented corn glucose, and those made by reacting AA with renewable alcohols, such as butanol from sugarcane [17,18]. The latter’s development employs bio-based monomers from renewable carbohydrate resources, such as cellulose, starch, and chitin. The resulting monomers are non-toxic, biocompatible, and biodegradable, making them ideal for the chemical industry. Furthermore, their complex structures offer substantial potential for manipulation [19,20,21].

**Scheme 1:**
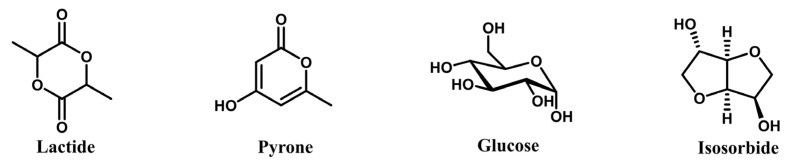
Carbohydrate feedstocks used for preparing bio-based PSAs.

Scheme 1 shows the carbohydrate feedstocks used for preparing bio-based PSAs, including lactide, pyrone, glucose, and isosorbide. To prepare high-performance PSAs, lactide with 2-ethyl hexyl acrylate (2-EHA) was used to initiate the polymerization with lactide and ε-caprolactone [22]. William et al. modified pyrone and copolymerized it with lauryl acrylate via reversible addition–fragmentation chain transfer (RAFT) polymerization to create PSAs having ABA triblock structures [23]. Furthermore, modified glucose can form segments with high glass transition temperature (*T*_g_) and thus improve the performance of PSA [24,25].

Isosorbide, produced through the dehydration of sorbitol, is a commercially available carbohydrate that provides stable batch production [26,27]. Compared to lactide and pyrone, isosorbide is more cost-effective. Additionally, its two hydroxyl groups can be used to prepare dimethyl isosorbide and isosorbide ester, which are applied as a solvent in cosmetics and a surfactant, respectively [28,29,30,31,32,33]. Owing to its two furan units, isosorbide exhibits high elasticity. The structural advantage renders isosorbide a strong candidate to replace glucose in bio-based polymer formulations [29,34,35]. Therefore, isosorbide is an ideal monomer for the production of high-performance bio-based polymers [36].

Isosorbide has also been employed to prepare bio-based PSAs. Jame et al. used isosorbide as a raw material to develop bio-based acrylic PSA through RAFT polymerization [37]. However, this adhesive was non-crosslinked and prone to aging, yellowing, and a loss of adhesion, resulting in a shorter service life than crosslinked adhesives. Baek et al. produced a bio-based PSA with isosorbide via UV-curing using a bifunctional and petrochemical-based crosslinking agent, which reduced the biomass content of the adhesive [38]. Additionally, a waterborne PSA was prepared using isosorbide methacrylate monomers through emulsion polymerization. However, its poor mechanical properties limited its application scope [39]. Although these findings successfully extend the application of isosorbide to PSAs, the limitations mentioned previously persist.

To address these challenges and expand the application of isosorbide in PSAs, functionalized isosorbide was selected and copolymerized with butyl acrylate (BA) and AA to synthesize a prepolymer of PSA. Isosorbide diacrylate ester (IDAE) was used as a crosslinker. Furthermore, a UV-curing process was adopted to create a novel bio-based PSA. To achieve a balance between adhesion and cohesion, the influence of the copolymer structure on the adhesion performance was studied via mechanical property tests, gel fraction analysis, and rheological tests.

## 2. Materials and Methods

### 2.1. Materials

BA, AA, and 2,2′-azobisisoheptonitrile (ABVN) used for the synthesis of the prepolymer were procured from Anhui Sunrise Technology Co., Ltd. (Anqing, China). Acryloyl chloride used for monomer synthesis was purchased from Heowns Biochem Technologies, LLC (Tianjin, China). Isosorbide and 2-methyl-4′-(methylthio)-2-morpholino-propiophenone were acquired from Shanghai Bepharm Science and Technology Co., Ltd. (Shanghai, China), which were used as a bio-based raw material and a photo initiator (PI), respectively. Dried tetrahydrofuran (THF), dichloromethane (DCM), and ethyl acetate (EA) were purchased from Shanghai Titan Scientific Co., Ltd. (Shanghai, China), as solvents for the reaction. All the above reagents have not been purified.

### 2.2. Characterization

The chemical structures of IA, IDAE, and the prepolymers were determined using a Bruker AVANCE AV III-400 NMR spectrometer (Bremen, Germany) with tetramethylsilane (TMS) as an internal standard in CDCl_3_.

FTIR (Fourier transform infrared) spectra in the wavelength range of 400–4000 cm^−1^ were collected using a Bruker Tensor FTIR 27 spectrometer (Coventry, UK) in attenuated total refraction (ATR) mode.

Gel permeation chromatography (GPC) was measured using a PL-GPC 50 instrument (Polymer Laboratories, Church Stretton, UK) equipped with a 5 μm guard column, a 5 μm mixed-D column, and a refractive index (RI) detector from Agilent Technology (Atlanta, GA, USA). THF was used as the eluent at a flow rate of 1 mL/min. The number-average molecular weight (*M*_n_) and dispersity (*Đ*) of the prepolymers were assessed at an injection concentration of 2 mg/mL. Number-average molecular weight and dispersity were measured using polystyrene (PS) as a standard.

To measure the gel fraction, 0.1 g of PSA was dissolved in 25 mL EA. After standing for 24 h, the residue was filtered through a 200-mesh copper screen and stabilized in a well-ventilated environment at room temperature to obtain its weight after desiccation. The gel fraction was calculated using the following equation:

(1) $$Gel(\%)=\frac{W_{b}}{W_{n}}\times 100\%$$

where *W_b_* is the mass of the PSA specimen, and *W_n_* is the mass of the insoluble residue after desiccation.

The *T*_g_ of PSAs was analyzed using a differential scanning calorimeter (DSC823e, Mettler Toledo, Schwarzenbach, Switzerland) in a nitrogen atmosphere. An indium standard was used for temperature calibration. To erase any prior thermal history, the sample was initially heated in an aluminum crucible at a heating rate of 20 °C/min from 80 °C and held for 2 min at 80 °C. After cooling from 80° to −80 °C at 20 °C/min and maintaining at −80 °C for another 2 min, the sample was reheated from −80 °C to 80 °C at 20 °C/min. The second heating run was used to determine the *T*_g_ of PSAs.

The adhesion performance of PSAs was assessed through 180° peel force tests, initial tack assessment, and persistent shear tests. Each test was performed thrice, and the results were averaged. According to GB/T 2792-2014 [40], the 180° peel force test was conducted to measure the force required to remove the tape from glass and stainless-steel plates at 25 °C. PSA was affixed to the test material with an area of 25 mm × 100 mm. A 2 kg rubber roller was used to roll the tape back and forth to prepare the samples for peel force testing. These samples were measured using a universal material testing machine (Instron, 3366, Norwood, MA, USA) at a loading speed of 300 mm/min.

The initial tack of PSA was evaluated according to GB/T 4852-2002 [41] by placing a steel ball and a polyethylene terephthalate (PET) film on a 20° inclined plate. The PET film was divided into a pre-loaded section (100 mm) and a PSA-coated bonding test section (100 mm). The initial tack was assessed by determining the maximum steel ball size to which the PSA effectively adhered.

Persistent shear tests were performed as per GB/T 4851-2014 [42], where PSA was attached to two standard steel plates pre-cleaned with methanol and subjected to two passes of a 2 kg rubber roller over a bonding area measuring 25 mm × 75 mm. The steel plates were mounted vertically, and a standard weight of 1 kg was hung from the free end of PSA to determine the time before adhesive failure.,

The rheological properties of PSA were tested using a strain-controlled rheometer (ARES-G2, TA Instrument, New Castle, DE, USA). This experiment investigated the flow behavior in parallel plate geometry (diameter: 25 mm, thickness: 1 mm) at 25 °C. The rheological behavior was measured when the scanning frequency varied from 0.1 to 100 rad/s.

### 2.3. Synthesis of Isosorbide-5-Acrylate (IA) and Isosorbide Diacrylate Ester (IDAE)

**Scheme 2:**
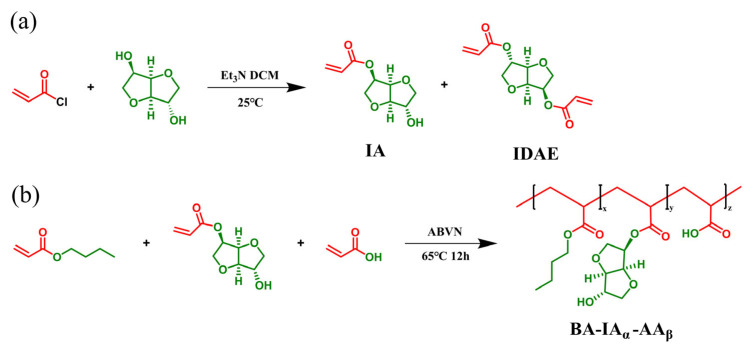
Synthetic routes of IA and IDAE (**a**) and the prepolymer (BA-IA_α_-AA_β_) (**b**).

IA and IDAE were synthesized following established procedures [38], as shown in Scheme 2a. Initially, 30 g (210 mmol) of IA was dissolved in a mixture containing 20 g (200 mmol) of triethylamine, 100 mL of dried THF, and 300 mL of DCM. A solution of acryloyl chloride in 75 mL of DCM was then slowly added to the mixture at 0 °C, followed by stirring at 25 °C for 24 h.

The reaction was quenched with distilled water (75 mL) and extracted thrice with DCM (300 mL). The combined organic layers were washed with a saturated saline solution and dried over Na_2_SO_4_. After filtering and concentrating under reduced pressure, a crude product was obtained. Column chromatography (SiO_2_) was used to purify IA and IDAE in a mixed solvent containing EA and DCM (volume ratio 1:10).

**Figure 1:**
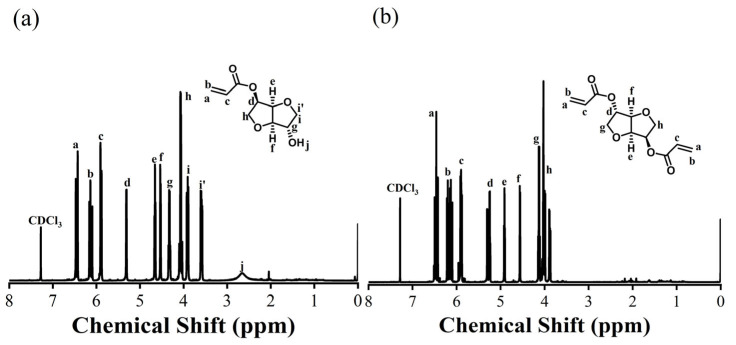
^1^H NMR spectra of IA (**a**) and IDEA (**b**).

The chemical structures of IA and IDAE were confirmed via nuclear magnetic resonance (NMR) spectroscopy, as shown in Figure 1.

IA: ^1^H NMR (400 MHz, chloroform-d) δ 6.44 (dd, J = 17.3, 1.3 Hz, 1H), 6.12 (dd, J = 17.3, 10.4 Hz, 1H), 5.89 (dd, J = 10.4, 1.3 Hz, 1H), 5.31 (d, J = 3.4 Hz, 1H), 4.65 (t, J = 4.9 Hz, 1H), 4.52 (d, J = 4.4 Hz, 1H), 4.32 (q, J = 5.8 Hz, 1H), 4.08–4.02 (m, 2H), 3.90 (dd, J = 9.5, 6.0 Hz, 1H), 3.58 (dd, J = 9.5, 6.0 Hz, 1H), and 2.24 (s, 1H).

IDAE: ^1^H NMR (500 MHz, chloroform-d) δ 6.44 (ddd, J = 18.9, 17.3, 1.3 Hz, 2H), 6.14 (ddd, J = 34.6, 17.3, 10.5 Hz, 2H), 5.95–5.83 (m, 2H), 5.32–5.20 (m, 2H), 4.89 (t, J = 5.0 Hz, 1H), 4.54 (d, J = 4.7 Hz, 1H), 4.05–3.99 (m, 2H), and 3.99–3.84 (m, 2H).

### 2.4. Synthesis of the Prepolymer

The prepolymer was synthesized using a radical polymerization method (Scheme 2b) [43], and the details of the monomers, initiator, and solvent are listed in Table 1. The selected monomers were introduced into a 250 mL three-necked, round-bottom flask and subjected to vacuum pumping to remove the residual gas. The monomers were then degassed with high-purity nitrogen gas and repeated thrice in a nitrogen atmosphere to ensure complete inertness. The reaction proceeded at 65 °C for 12 h, after which the solvent was removed through vacuum distillation.

The resulting prepolymer was designated as BA-IA_α_-AA_β_, where α and β were expressed as the ten-fold molar ratio of IA to BA and AA to BA, respectively.

### 2.5. Preparation of PSAs

**Figure 2:**
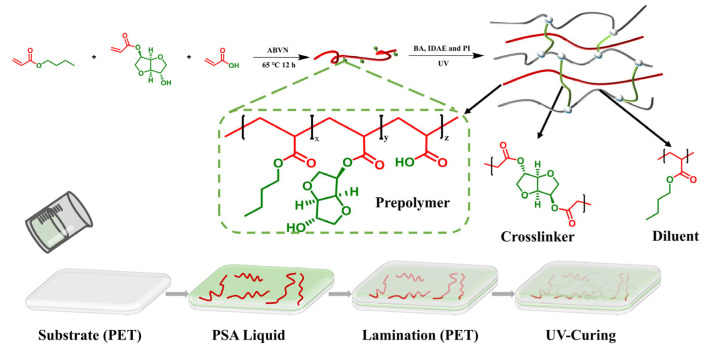
Synthetic mechanism of PSA and schematic of the preparation process of PSAs.

An appropriate amount of BA was added to a 20 mL plastic cup as a diluent to reduce the viscosity of the prepolymer during coating and ensure the optimal fluidity of the slurry. Then, 2-methyl-4’-(methylthio)-2-morpholino-propiophenone and IDEA were introduced to the polymerization system, which were used as a photo initiator and as a synthetic, bifunctional crosslinker to enhance the structural integrity of the adhesive, respectively. Finally, all comments of PSA in the cup were mixed in a Hauschild Speedmixer (DAC 150R, Hauschild, Hamm, Germany) at a rotation speed of 3000 rpm for 5 min. The resulting PSA was designated as BA-IA_α_-AA_β_-γ, in which “γ” represented the weight ratio of the crosslinker to the diluent. Detailed information on these components is provided in Table 2. Figure 2 shows the synthetic mechanism of PSA.

### 2.6. UV-Curing

A blend of PSA adhesive solution was applied to a thin PET substrate, whose thickness was controlled precisely at 65 μm using a precision coating machine operating at a speed of 2 cm/s. After covering another layer of PET film, a PET tape with a stratified “sandwich” structure was formed. To exclude oxygen and ensure an oxygen-free environment for the PSA tape, roller compression was applied before the UV-curing. The final cured thickness of the adhesive layer was approximately 30 ± 2 μm, as shown in Figure 2. PSA tapes were cured for 4 min at a wavelength of 365 nm on a UV-curing machine (Shenzhen Huafang photoelectric Co., LTD., Shenzhen, China) equipped with a low-pressure mercury lamp at room temperature. The UV-curing parameters were monitored using a UV light-energy meter to ensure experimental consistency (Table 3).

## 3. Results and Discussion

### 3.1. Polymerization of Prepolymers

**Figure 3:**
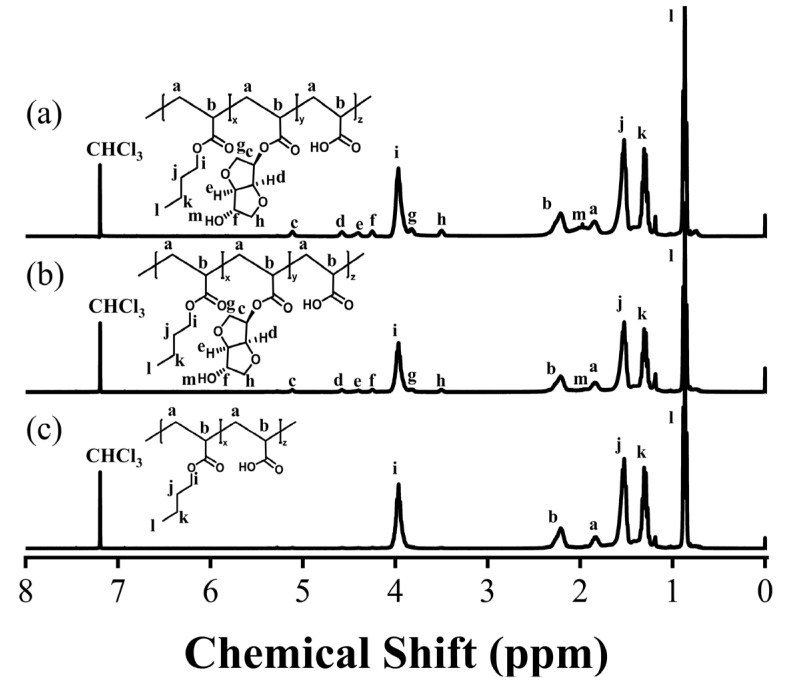
^1^H NMR spectra of BA-IA_0_-AA_2_ (**a**), BA-IA_5_-AA_2_ (**b**), and BA-IA_10_-AA_2_ (**c**).

**Figure 4:**
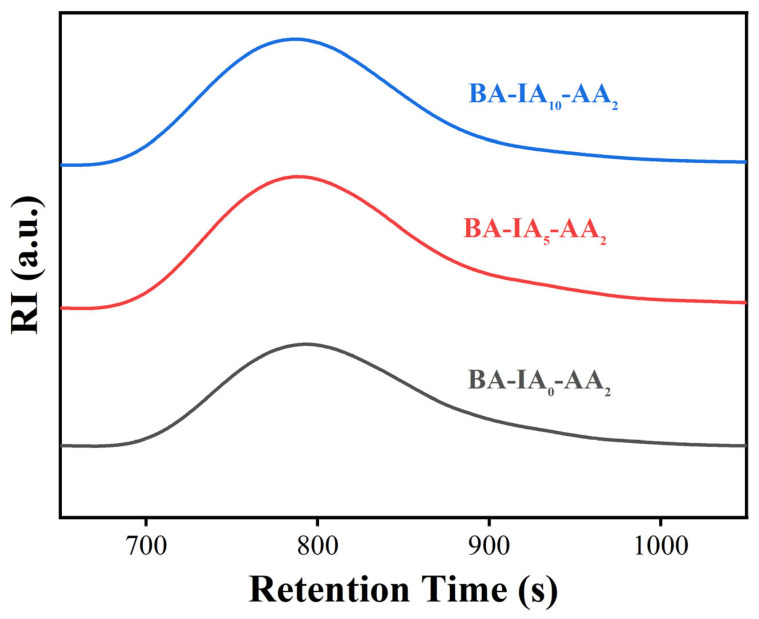
GPC chromatograms of the prepolymers.

**Figure 5:**
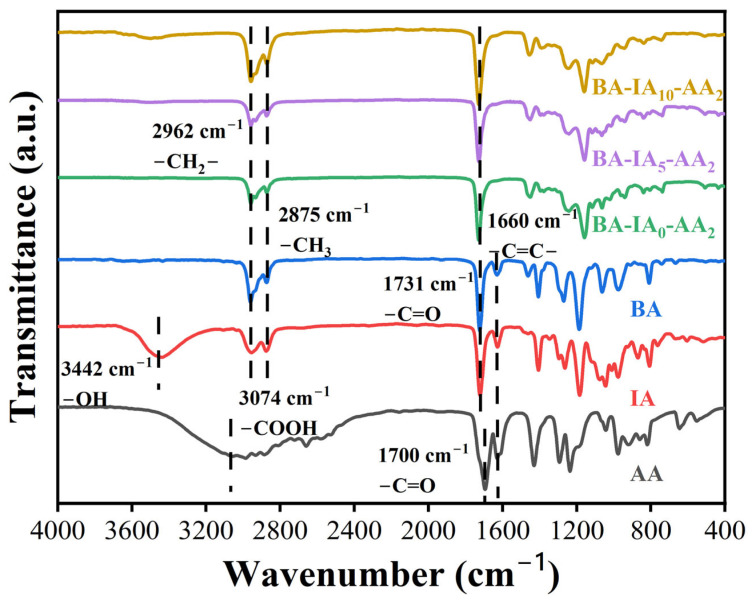
FTIR spectra of the prepolymers, BA, AA, and IA monomers.

The chemical structures of the prepolymers were confirmed via NMR spectroscopy. As shown in Figure 3, the characteristic peak of IA appears in the NMR spectrum of the prepolymer, whereas the characteristic peak of vinyl disappears, indicating that the prepolymer was synthesized with a high conversion rate. The molecular weights and distributions were determined via GPC, as detailed in Table 1 and Figure 4. The molar quantity of the initiator (ABVN) remains constant throughout the study, resulting in a prepolymer with a relatively high molecular weight and a uniform molecular weight distribution (Table 1). FTIR spectroscopy has been widely used to confirm the chemical structure of adhesives [44,45,46]. As shown in Figure 5, in the spectrum of IA, a distinct −OH absorption band appears at 3442 cm^−1^. For AA, the absorption peaks corresponding to −COOH and −C=O are identified at 3074 cm^−1^ and 1700 cm^−1^, respectively. Additionally, absorption peaks at 2692 cm^−1^ and 2875 cm^−1^ are attributed to the −CH_2_− and −CH_3_ groups in the prepolymers BA and IA, respectively. Characteristic peaks for the −C=O groups appear at 1731 cm^−1^ in both prepolymers BA and IA. Notably, the C=C peaks around 1660 cm^−1^ shown in BA, AA, and IA monomers are absent from the spectra of the prepolymers, indicating that these monomers underwent copolymerization with a high conversion rate.

### 3.2. Gel Fraction

**Figure 6:**
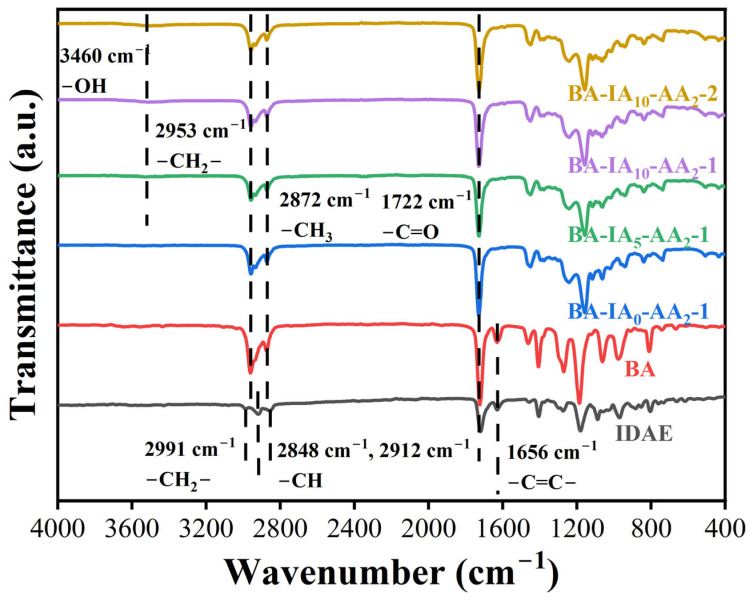
FTIR spectra of the PSAs, IDAE, and BA samples.

In this study, several acrylic PSAs exhibit crosslinked structures. In addition, the crosslinking degree significantly influences the adhesion performance of PSAs. The UV-curing samples were analyzed via FTIR. As shown in Figure 6, in the crosslinked samples, the absorption peaks of −OH, −CH_2_−, CH_3_, and −C=O appear at 3460 cm^−1^, 2953 cm^−1^, 2872 cm^−1^, and 1722 cm^−1^, respectively. The absence of C=C peaks for both the crosslinker (IDAE) and the diluent (BA) is consistent with the reaction of BA with IDAE’s C=C, indicating that crosslinked networks are formed in the presence of the photo initiator.

**Figure 7:**
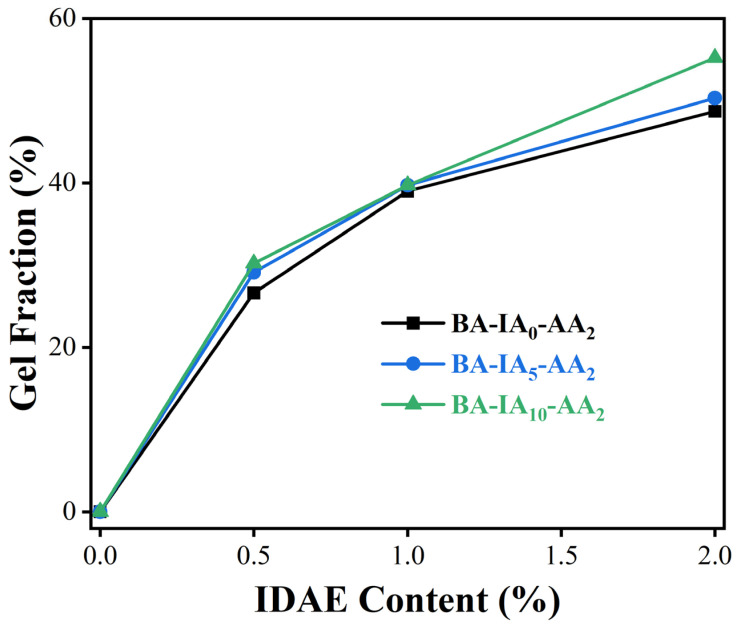
Gel fractions of PSAs with different IDAE concentrations.

In this work, the crosslinking degree was assessed using a gel fraction test. As shown in Figure 7, the gel fractions of bio-based PSAs increase from 0% to 48.7%, 50.3%, and 55.2%, respectively, as the crosslinker content increases, i.e., tightly crosslinked networks with a higher crosslinker content are formed at the same prepolymer condition. When the crosslinker content remains constant, the gel fractions of the different prepolymers remain consistent, demonstrating the stability of the UV-curing process. This stability is further corroborated by data from ultraviolet energy meters (Table 2).

### 3.3. Adhesion Performance of PSAs

In this study, 180° peel force, initial tack, and persistent shear tests were used to evaluate the adhesion performance of PSAs according to relevant standards. High-performance PSAs need to exhibit a balance between adhesion and cohesion despite the inherent conflict between these properties [26,47].

**Figure 8:**
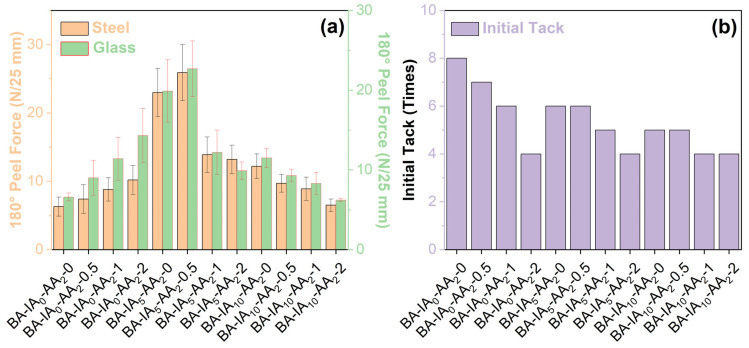
The 180° peel force (**a**) and initial tack numbers (**b**) of PSAs.

The rolling ball method was used to assess the initial adhesion, as detailed in the experimental section, revealing that PSAs exhibit initial adhesion to stainless-steel balls. During the rolling process, the energy loss is primarily due to the work required to disrupt and re-establish the bonding interface (*W*_a_). According to a theoretical formula, the initial tack of PSA decreases with a reduction in *W*_a_ [48]. The initial tack numbers for PSAs with the same crosslinker content but different IA contents and those with the same IA content but different crosslinker contents show that the addition of IA restricts polymer chain movement. An increase in the crosslinking density reduces the surface fluidity, *W*_a_, and the initial tack number (Table 4 and Figure 8).

**Figure 9:**
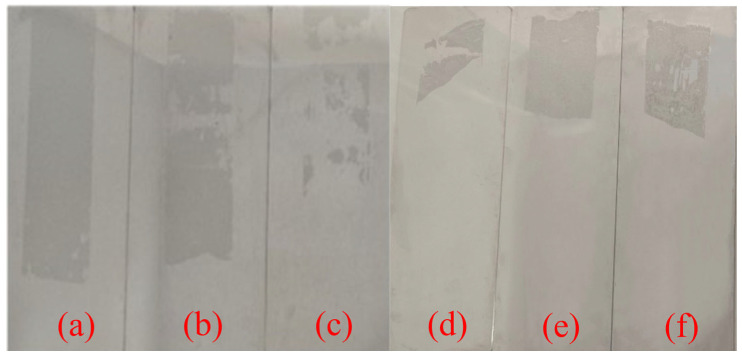
Cohesion failure patterns of BA-IA_0_-AA_2_-0 (**a**), BA-IA_0_-AA_2_-0.5 (**b**), BA-IA_0_-AA_2_-1 (**c**), BA-IA_0_-AA_2_-2 (**d**), BA-IA_5_-AA_2_-0 (**e**), and BA-IA_5_-AA_2_-0.5 (**f**).

To conduct a detailed analysis of the peel strength of PSA, this study employed stainless-steel and glass plates as test substrates to perform 180° peel force tests on PSA. The results in Table 4 and Figure 8 demonstrate that with an increase in the crosslinker content of the unmodified prepolymer (without IA), the peel force of PSAs gradually increases on both stainless steel and glass. Six of these samples exhibit cohesion failure after peeling (Figure 9) because the absence of the rigid unit IA leads to insufficient interatomic forces [35], which compromises the adhesion in the adhesive material.

As the amount of crosslinker (IDAE) increases, the interatomic forces of PSA rise gradually and thus increase the peel force against the steel plate from 6.3 N/25 mm to 10.2 N/25 mm and against the glass from 6.6 N/25 mm to 14.3 N/25 mm, as depicted in Figure 8. For PSAs containing 5% IA, the 180° peel force first increases, followed by a sharp decline. Notably, in this sample set, PSAs with 0 and 0.5% IDAE display a cohesion failure mode, whereas the others demonstrate an adhesive failure mode. Regarding BA-IA_5_-AA_2_-0 and BA-IA_5_-AA_2_-0.5, their cohesive force remains lower than the adhesive force. Because IA enhances the rigidity of PSAs, the crosslinker with the concentration of 1 and 2% leads to a transition from cohesion failure to adhesive failure, where the adhesive force exceeds the cohesion force on the adhered surface. However, owing to the reduced fluidity and wetting ability caused by the increased IA and crosslinker contents on the adhered surface, a downward trend is observed in the 180° peel force. Furthermore, when 10% IA is introduced to this batch of PSAs, the 180° peel force decreases as the crosslinker content increases due to the strong cohesion induced by the adequate IA content. At this juncture, the magnitude of the cohesion force exceeds that of the adhesive force and PSA manifests cohesion failure, thereby causing a decrease in the adhesive force with an increase in the crosslinker content. Furthermore, for PSAs with the same crosslinker content, an increase in the IA content leads to an initial increase in persistent time, followed by a decline in the 180° peel force (Table 4 and Figure 8). This trend is associated with the increase in *T*_g_ of prepolymers owing to the elevation of the rigid unit (IA) and crosslinking density, as depicted in Figure 6. During cohesion failure, the incorporation of IA significantly enhances the peel strength. However, a higher *T*_g_ indicates that the chain of PSA is “more rigid” and less favorable for wetting on the adhesive surface during adhesive failure, giving rise to a downward tendency in the 180° peel force.

A persistent shear test was conducted to assess the holding adhesion of PSAs. This test measured the time required for the adhesion to fail under a specific weight. The longer the hanging intervals, the better the adhesion performance. As shown in Table 4, the non-crosslinked PSA in the control group (BA-IA_0_-AA_2_-0) maintains adhesion for only 5 min under a 1 kg load, primarily due to insufficient interatomic forces and thus cohesion failure. Increasing the crosslinker concentration from 0 to 2% leads to the formation of a more robust network structure, extending the adhesion time to 896 min. Additionally, the persistent shear results at the same crosslinker content with varying IA contents (5% and 10%) show similar trends, and the adhesion times of BA-IA_5_-AA_2_-2, BA-IA_10_-AA_2_-1, and BA-IA_10_-AA_2_-2 exceed 10,000 min. A higher IA content improves the persistent shear because rigid IA is favorable for enhancing cohesion. These findings demonstrate that a higher crosslinking degree and IA content contribute to stronger cohesion and thus significantly longer adhesion times under stress.

The test results of the initial tack, 180° peel force, and persistent shear indicate that the moderate addition of IA and appropriate crosslinker significantly enhance the adhesion performance of PSAs. As presented in Table 5, the bio-based PSAs produced in this study demonstrate superior adhesion properties compared to PSAs modified by cellulose nanocrystal (CNC) and functionalized CNC (fCNC) with 3-methacryloxypropyltrimethoxysilane [49]. Based on the experimental data, BA-IA_0_-AA_2_-1, BA-IA_5_-AA_2_-1, BA-IA_10_-AA_2_-1, and BA-IA_10_-AA_2_-2 exhibit the best balance between adhesion and cohesion, which were selected for further investigation.

### 3.4. Differential Scanning Calorimetry

**Figure 10:**
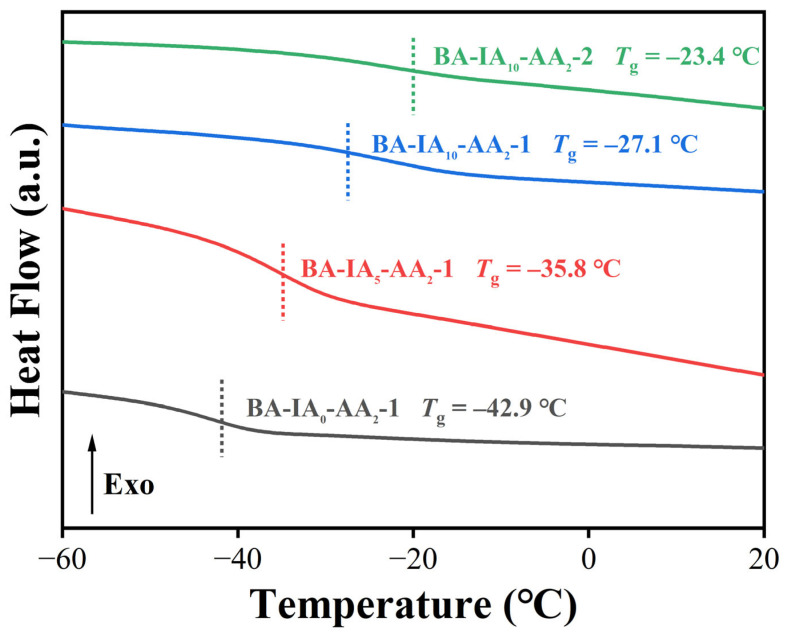
DSC heating scan of the selected PSAs.

*T*_g_ is a critical factor in assessing the suitability of a polymer system for PSA application; an appropriate *T*_g_ tends to enhance the pressure-sensitive properties, particularly the peel strength. The *T*_g_ of PSA depends on its composition. DSC is a useful tool to determine the *T*_g_ of polymeric materials [50,51,52]. Figure 10 shows the DSC curves of BA-IA_0_-AA_2_-1, BA-IA_5_-AA_2_-1, BA-IA_10_-AA_2_-1, and BA-IA_10_-AA_2_-2. BA-IA_0_-AA_2_-1 exhibits a *T*_g_ of −42.9 °C, primarily due to the presence of long “soft segments” within the polymer chains. In contrast, the rigid structure of IA with two furan units tends to increase *T*_g_. With the addition of 5% IA, the *T*_g_ of PSA (BA-IA_5_-AA_2_-1) increases to −35.8 °C. At an IA content of 10%, as the crosslinker concentration increases from 1 to 2% (BA-IA_10_-AA_2_-1 to BA-IA_10_-AA_2_-2), the *T*_g_ raises from −27.1 to −23.4 °C due to the reduction in free volume and the restriction of molecular chain movement, as well as the decrease in flexibility.

### 3.5. Rheological Analysis

The adhesion characteristics of PSAs strongly depend on their viscoelastic properties. For a PSA, adhesion relates to viscous behavior for generating tack, while cohesion relates to elasticity for removing clearly from the adherend and sustaining loads [47]. Therefore, the balance of adhesion and cohesion is crucial in PSA applications.

The viscoelastic behavior of PSAs was evaluated through dynamic mechanical or rheological testing. PSAs interact with substrates at low frequencies. According to the “Dahlquist” criterion, PSAs can function when the storage modulus (G′) is less than 3.3 × 10^5^ Pa under operating temperature and low-frequency conditions [53]. Additionally, Chang et al. introduced the “viscoelastic window” concept, estimating the practical adhesive properties of various PSAs across different environmental conditions [54]. Based on these approaches, an advanced rheological technique was employed to measure G′ and the loss modulus (G″), which indicates the elastic and viscous characteristics of the material, respectively.

**Figure 11:**
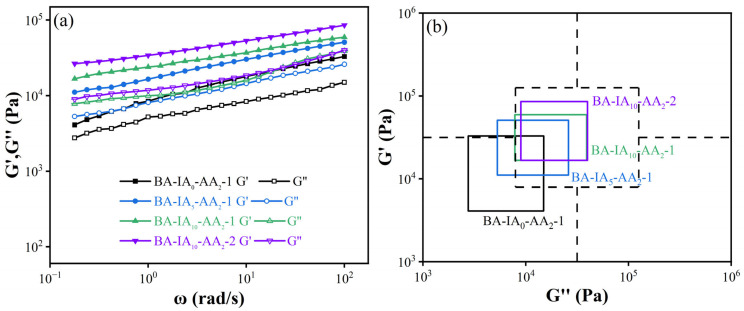
Plots of storage and loss modulus versus frequency (**a**) and viscoelastic windows (**b**) of PSAs.

As shown in Figure 11a, G′ surpasses G″ for all PSAs, indicating that their elastic properties are superior to viscous behaviors. With the incorporation of IA and the enhanced crosslinking strength, both G′ and G″ increase, indicating elevated molecular chain rigidity. Based on the G′ and G″ values at the corresponding frequencies, the viscoelastic windows are constructed. As shown in Figure 11b, the viscoelastic window is divided into five zones, each corresponding to a different type of PSA. The gradual addition of IA improves the viscoelastic properties of PSAs, resulting in the movement closer to the central region, which is suitable for general applications. Furthermore, when the IA content remains constant but the crosslinker concentration increases from 1 to 2%, the viscoelastic window shifts with a similar trend. In summary, the viscoelastic performance of PSAs can be effectively enhanced by tuning the IA and crosslinker concentrations and thus meets the requirements for general PSA applications.

## 4. Conclusions

In this study, bio-based acrylic PSAs were successfully synthesized with two isosorbide-based raw materials, IA and IDEA, which were used as the hard segment and the crosslinker, respectively. The correlation between the structure and performance of PSAs was thoroughly investigated. FTIR results revealed that these components are effectively integrated into prepolymer chains. The introduction of rigid IA and crosslinkers increases the *T*_g_ of PSAs because the tightly crosslinked networks restrict molecular chain movements. When adding 5% IA, the *T*_g_ of BA-IA_0_-AA_2_-1 increases from −42.9 °C to −35.8 °C. For BA-IA_10_-AA_2_, the *T*_g_ increases from −27.1 °C to −23.4 °C when the IDEA concentration rises from 1% to 2%. The inclusion of IA and IDEA significantly improves both the peel force and shear strength. Furthermore, the peel force of PSAs increases in IDEA concentration. The persistent time of PSAs increases in the concentration of both IA and IDEA. Under optimal conditions, the 180° peel force of BA-IA_5_-AA_2_-0.5 reached 25.9 N/25 mm, while the longest hanging time exceeds 10,000 min. Overall, BA-IA_5_-AA_2_-1 with a peel force of 13.9 N/25 mm and a persistent time of 6820 min demonstrates the best balance between adhesion and cohesion. Due to its excellent performance, the developed bio-based PSA exhibits considerable potential to replace petroleum-based PSAs.

## Figures and Tables

**Table 1:** Compositions and polymerization data of prepolymers_._

Sample	BA (mmol)	IA (mmol)	AA (mmol)	ABVN (mmol)	EA (wt%)	*M*_n_ (kg/mol)	*Đ*
BA-IA_0_-AA_2_	156	0	3.1	0.195	65	174	2.68
BA-IA_5_-AA_2_	156	7.8	3.1	0.195	65	193	2.66
BA-IA_10_-AA_2_	156	15.6	3.1	0.195	65	186	2.74

**Table 2:** Compositions of PSAs.

Sample	Prepolymer (g)	Diluent (g)	PI (mg)	IDAE (mg)
BA-IA_0_-AA_2_-0	5	2.5	2.64	0
BA-IA_0_-AA_2_-0.5	5	2.5	2.64	12.5
BA-IA_0_-AA_2_-1	5	2.5	2.64	25
BA-IA_0_-AA_2_-2	5	2.5	2.64	50
BA-IA_5_-AA_2_-0	5	2.5	2.64	0
BA-IA_5_-AA_2_-0.5	5	2.5	2.64	12.5
BA-IA_5_-AA_2_-1	5	2.5	2.64	25
BA-IA_5_-AA_2_-2	5	2.5	2.64	50
BA-IA_10_-AA_2_-0	5	2.5	2.64	0
BA-IA_10_-AA_2_-0.5	5	2.5	2.64	12.5
BA-IA_10_-AA_2_-1	5	2.5	2.64	25
BA-IA_10_-AA_2_-2	5	2.5	2.64	50

**Table 3:** UV-curing parameters for PSA curing.

Sample	*E* ^a^ (mJ/cm^2^)	*P*_MAX_^b^ (mW/cm^2^)	*T*_MAX_^c^ (°C)
BA-IA_0_-AA_2_-0	1801.2	7.3	29.4
BA-IA_0_-AA_2_-0.5	1789.3	7.2	29.3
BA-IA_0_-AA_2_-1	1814.6	7.1	29.5
BA-IA_0_-AA_2_-2	1844.1	7.3	29.4
BA-IA_5_-AA_2_-0	1763.5	7.0	28.7
BA-IA_5_-AA_2_-0.5	1712.5	7.2	29.4
BA-IA_5_-AA_2_-1	1789.5	7.1	29.1
BA-IA_5_-AA_2_-2	1710.3	7.1	29.1
BA-IA_10_-AA_2_-0	1713.7	7.0	29.2
BA-IA_10_-AA_2_-0.5	1745.4	7.2	29.1
BA-IA_10_-AA_2_-1	1715.0	7.1	29.4
BA-IA_10_-AA_2_-2	1770.7	7.1	30.2

^a^ The absorption of energy during UV-curing. ^b^ The maximum power absorbed during UV-curing. ^c^ The maximum temperature detected during UV-curing.

**Table 4:** Adhesive properties of PSAs.

Sample	Persistent Time (min)	*T*_g_ (°C)
* BA-IA_0_-AA_2_-0	5	
* BA-IA_0_-AA_2_-0.5	76	
* BA-IA_0_-AA_2_-1	376	−42.9
* BA-IA_0_-AA_2_-2	896	
* BA-IA_5_-AA_2_-0	21	
* BA-IA_5_-AA_2_-0.5	4517	
BA-IA_5_-AA_2_-1	6820	−35.8
BA-IA_5_-AA_2_-2	>10,000	
BA-IA_10_-AA_2_-0	391	
BA-IA_10_-AA_2_-0.5	6890	
BA-IA_10_-AA_2_-1	>10,000	−27.1

* Cohesion failure after peeling.

**Table 5:** Adhesion properties of BA-IA_5_-AA_2_-1.

Sample	Initial Tack (Times)	Persistent Time (min)	180° Peel Force (N/25 mm)
BA-IA_5_-AA_2_-1	5	6820	13.9
CNC-1 ^a^ [49]	24	4020	6.5
fCNC-1 ^b^ [49]	27	4200	13.4

^a^ PSA with 1 wt% of CNC. ^b^ PSA with 1 wt% functionalized CNC.

## Data Availability

Data are contained within the article.
